# Whole Exome Sequencing Identifies a Novel Homozygous Missense Mutation in the CSB Protein-Encoding *ERCC6* Gene in a Taiwanese Boy with Cockayne Syndrome

**DOI:** 10.3390/life11111230

**Published:** 2021-11-14

**Authors:** Ching-Ming Lin, Jay-How Yang, Hwei-Jen Lee, Yu-Pang Lin, Li-Ping Tsai, Chih-Sin Hsu, G. W. Gant Luxton, Chih-Fen Hu

**Affiliations:** 1 Department of Pediatrics, Tri-Service General Hospital, National Defense Medical Center, Taipei 11490, Taiwan; wwwwhccps@mail.ndmctsgh.edu.tw; 2 Department of Pediatrics, Kaohsiung Armed Forces General Hospital, Kaohsiung 80284, Taiwan; 3 Center for Applied Structural Discovery, Biodesign Institute, Arizona State University, Tempe, AZ 85281, USA; jyang62@asu.edu; 4 Department of Biochemistry, National Defense Medical Center, Taipei 11490, Taiwan; hjlee@ndmctsgh.edu.tw; 5 Department of Radiology, Tri-Service General Hospital, National Defense Medical Center, Taipei 11490, Taiwan; gardneri@mail.ndmctsgh.edu.tw; 6 Department of Pediatrics, Taipei Tzu Chi Hospital, Buddhist Tzu Chi Medical Foundation, New Taipei 23142, Taiwan; tsaiped@tzuchi.com.tw; 7 Genomics Center for Clinical and Biotechnological Applications of Cancer Progression Research Center, National Yang Ming Chiao Tung University, Taipei 11221, Taiwan; jc858720@nycu.edu.tw; 8 Department of Molecular and Cellular Biology, University of California-Davis, Davis, CA 95616, USA

**Keywords:** Cockayne syndrome B, progeroid, *ERCC6*, whole exome sequencing, transcription-coupled nucleotide excision repair (TC-NER)

## Abstract

Background: Cockayne syndrome (CS) is a rare form of dwarfism that is characterized by progressive premature aging. CS is typically caused by mutations in the excision repair cross-complementing protein group 6 (*ERCC6*) gene that encodes the CS group B (CSB) protein. Using whole exome sequencing, we recently identified a novel homozygous missense mutation (Leu536Trp) in CSB in a Taiwanese boy with CS. Since the current database (Varsome) interprets this variant as likely pathogenic, we utilized a bioinformatic tool to investigate the impact of Leu536Trp as well as two other variants (Arg453Ter, Asp532Gly) in similar articles on the CSB protein structure stability. Methods: We used iterative threading assembly refinement (I-TASSER) to generate a predictive 3D structure of CSB. We calculated the change of mutation energy after residues substitution on the protein stability using I-TASSER as well as the artificial intelligence program Alphafold. Results: The Asp532Gly variant destabilized both modeled structures, while the Leu536Trp variant showed no effect on I-TASSER’s model but destabilized the Alphafold’s modeled structure. Conclusions: We propose here the first case of CS associated with a novel homozygous missense mutation (Leu536Trp) in CSB. Furthermore, we suggest that the Asp532Gly and Leu536Trp variants are both pathogenic after bioinformatic analysis of protein stability.

## 1. Introduction

Cockayne syndrome (CS; MIM# 133540, 216400), also known as Neill–Dingwall syndrome, is a rare autosomal recessive neurodegenerative disorder that is characterized by progressive growth failure, microcephaly, global developmental delay, cutaneous photosensitivity, and premature pathological aging [1]. According to the presentation of clinical phenotype and the disease severity, CS can be described as CS type I, CS type II, CS type III, cerebrooculofacioskeletal syndrome (COFS), and UV-sensitive syndrome (UVSS) [2]. As a result of the wide clinical variability and the difficulty of early CS diagnosis, molecular genetic testing could be a useful tool to confirm the diagnosis efficiently. CS has been divided into two categories according to the mutation of the gene: (1) CS type A (CSA), which is caused by a mutation in the *ERCC8* gene located on chromosome 5q12.1, and (2) CS type B (CSB), which is caused by a mutation in the *ERCC6* gene located on chromosome 10q11. CSB accounts for 62–75% of all CS cases [1,3].

The *ERCC6* gene encodes a protein of 1493 amino acids with seven ATPase motifs known as CSB [4]. This protein belongs to the SWI2/SNF2 family and is engaged in nucleotide excision repair (NER) as well as base excision repair (BER). The transcription coupled repair (TCR) pathway, a sub-pathway of NER, preferentially removes UV-induced DNA lesions from the transcribed strand of actively transcribed regions of the genome. In addition, several studies disclosed that CSB is also involve in BER, which repairs oxidative stress-induced DNA damage caused by endogenous and exogenous sources [5]. After nearly 60 years of research in the NER field regarding progeroid CS, it is largely accepted that CS is caused by a combination of altered gene transcription, redox imbalance, metabolic disturbance, and DNA repair defects. However, the contributions and importance of these mechanisms for CS remain unknown [6].

Here, we present a case of CS type I with a novel homozygous missense mutation (c.1607T>G, p.Leu536Trp) in the *ERCC6* gene detected by whole exome sequencing (WES) from a Taiwanese family. Leu536Trp was previously identified as one of the variants in the form of compound heterozygous mutation published in two articles (Asp532Gly and Leu536Trp; Arg453Ter and Leu536Trp) [7,8]. To our knowledge, this missense mutation has not been reported in the form of homozygosity before. Furthermore, this mutation is classified as likely being a pathogenic variant by the American College of Medical Genetics and Genomics (ACMG) guideline [9], and we calculated the mutation energy of this variant on predictive protein structures to give objective and indirect evidence on its impact on CSB structural stability.

## 2. Materials and Methods

### 2.1. Human Subjects

This study recruited three human subjects, including one proband and his parents of Taiwanese ancestry. All subjects (or legal guardians) gave their written informed consent for participation, and the study was approved by the Institutional Review Board of the Tri-Service General Hospital (IRB# NO. C202105054). Detailed clinical information was obtained from corresponding clinicians and medical records.

### 2.2. Purification of Genomic DNA from Isolated Human Blood Leukocytes

Genomic DNA was purified from human leukocytes using the MagPurix^®^ Blood DNA Extraction Kit LV and run in the MagPurix 24^®^ Nucleic Acid Extraction System (Labgene Scietific^®^, SA, Châtel-Saint-Denis, Switzerland) following the instructions provided by the manufacturer.

### 2.3. WES, Sanger Sequencing, and Database Interpretation

WES of the proband was performed by the Beijing Genomics Institute^®^, and the result was interpretated by Dr. Pi-Lin Sung, a clinical geneticist at Shuang Ho hospital in Taiwan. Sequencing reads were mapped to the GRCh37/hg19 reference genome by using the Burrows–Wheeler algorithm [10]. The WES test report revealed the presence of a missense mutation in the *ERCC6* gene due to a homozygous point mutation at the genomic level [NM_000124.2: c.1607T>G; p.Leu536Trp].

Sanger sequencing of the proband and his parents was performed to determine if this mutation was inherited from the parents or were caused by a sporadic event. The DNA encoding portion of the *ERCC6* gene was PCR amplified from the genomic DNA using the following primer pairs: *ERCC6* (c.1607T>G, p.Leu536Trp) F: CTGCCCTACAGCTCCATT and R: TCCACCATTTGCCATTTT (PCR product: 386 bp). The resulting PCR products were purified using QIAquick PCR Purification Kit (Qiagen^®^) and then subjected to Sanger sequencing (Genomics^®^, Taipei, Taiwan).

Several bioinformatics databases were used to interpret the pathogenicity of this mutation in the *ERCC6* gene, including Varsome, Uniport, ClinVar, ClinVar Miner, and dbSNP. Furthermore, the allele frequency of the variant was investigated in the Taiwan BioBank as a local database and in the Genome Aggregation Database (gnomAD) as a global database.

### 2.4. Multiple Sequence Alignment of CSB

The ATP-binding domain amino acids of CSB (Arg453, Asp532, and Leu536) from homologous genes in mammals, birds, reptiles, and bony fishes was analyzed by EMBL-EBI-Multiple Sequence Alignment, Clustal Omega (CLUSTAL O 1.2.4) [11].

### 2.5. Predictive Three-Dimensional Models of CSB

We utilized the iterative threading assembly refinement (I-TASSER) server [12] to predict the partial (aa: 440–550) and full-length (aa: 1–1493) sequences of CSB. The I-TASSER server is an integrated platform for automated protein structure and function prediction based on the sequence-to-structure-to-function paradigm. Based on the amino acid sequence, it generates three-dimensional (3D) atomic models from multiple threading alignments and iterative structural assembly simulations. We labeled the mutated amino acids (Leu536) found in our case as well as the other two mutated amino acids (Arg453, Asp532) found in similar cases from related articles [7,8].

Meanwhile, we superimposed the CSB structure predicted by I-TASSER on an independent one generated by the AlphaFold Protein Structure Database (https://alphafold.ebi.ac.uk/entry/Q03468, last updated on 1 July 2021) [13] in partial (488–1011) and full-length (1–1493) respectively by the root-mean-square deviation (RMSD) method [14]. AlphaFold is another computational method that can be used to predict protein structures with atomic accuracy, even in cases which no similar structure is known. Hence, we can evaluate and compare the similarity of these two predictive models and then utilize the results in additional advanced bioinformatic analyses.

Finally, we compared the predictive CSB structure generated by I-TASSER with current known crystal structures from Newman’s model (90–158) (PDB code: 4CVO) and Takahashi’s model (1–76 and 1400–1493), respectively [15] (PDB code: 6A6I).

### 2.6. Evaluating the Effect of the Change of Mutation Energy Caused by Mutations on the Stability of CSB

The effect of residue substitution on the stability of CSB determined from the two predictive models from I-TASSER and Alphafold using the software of Discovery studio visualizer version v19.1.0.18287 (BIOVIA, San Diego, CA, USA) [16]. As for the calculation of the change in mutation, the energy is normalized to wild-type CSB. On the other hand, the impact of the Arg453Ter variant on CSB stability could not be calculated by this method.

## 3. Results

### 3.1. Case Presentation

The proband, a 3-year-old boy, is the only child of non-consanguineous parents. The parents are of Taiwanese origin. To date, the father, a 44-year-old male, and the mother, a 41-year-old female, do not have any neurological disorders or medical illnesses. In addition, no other members in paternal or maternal families have a similar phenotype or clinical presentation. Regarding prenatal and birth history, amniocentesis for a karyotype test was performed at the 18th week of gestational age because of advanced maternal age (37-year-old), which showed 46 chromosomes, XY. The child was born at 39 and 2/7 weeks of gestational age by normal spontaneous delivery. His birth body weight was 2780 g (<10th percentile). His head circumference and body length were reported as normal at birth.

**Figure 1:**
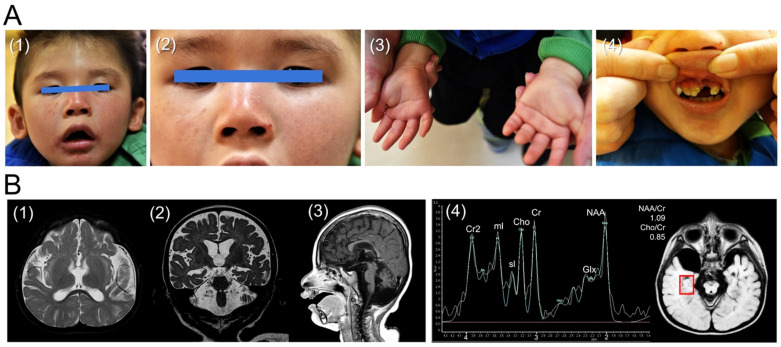
The photos and brain MRI of the proband. (**A**) Photos of the male proband at 3 years old. (1) General appearance of the face, which reveals a thin pointy nose and a mildly wizened face; (2) Zoom-in of the face shown in (1) showing sun-exposed skin photosensitivity as revealed by increased pigmentation over nose and cheek area, which is one of the typical characteristics of CS patients [1]; (3) bilateral single palmar crease; (4) severe dental caries. (**B**) Brain MRI of the proband performed at 2 years and 6 months old. (1) Axial short tau inversion recovery (STIR) images show generalized cerebral atrophy with ventricular dilatation, increased signal intensity in the subcortical white matter and external capsules. (2) Coronal T2-weighted images demonstrate hyperintensities in centrum semiovale and internal capsules. (3) Midline sagittal T1-weighted images reveal a thin corpus callosum and moderate atrophy of the brain stem and cerebellum, resulting in an enlarged cisterna magna. (4) MRS showing a decreased NAA/Cr ratio (1.09) in the gray matter of the medial temporal lobe. These combined neuroradiologic findings can assist in the diagnosis of CS.

He was initially referred to our hospital at the chronological age of 1 year and 8 months because of development delay. The patient had appropriate milestones in the first six months of life, but he was not able to crawl until 10 months of age. He could not walk independently at the chronological age of 1 year and 8 months, and he could only pronounce one or two meaningful words (e.g., “dada” and “mama”) at this age. Furthermore, postnatal growth retardation (<5th percentile), disproportionate microcephaly, dental caries, wizened face with prominent nasolabial folds, mild skin pigmentation, and a single palmar crease were also found during his physical examinations (Figure 1A). A neurological examination revealed that he exhibited alert consciousness, occasional eye contact, hypotonia of four limbs with preserved tendon reflexes, and a lack of meaningful words production.

Laboratory investigations for metabolic disorders, including electrolytes, thyroid, renal, and liver function, blood gas analysis, serum ammonia, lactate, amino acid tandem mass spectrometry, urine organic acid, and neuronal ceroid lipofuscinoses were examined with the results being all within normal reference. As a result of this significantly impaired language development, an auditory brainstem response (ABR) was applied, which revealed bilateral sensorineural hearing loss. Moreover, a fundoscopic exam discovered bilateral grade 1 optic atrophy.

Awake and sleep electroencephalography (EEG), nerve conduction velocity examination (NCV), and brain magnetic resonance imaging (MRI) were performed at the chronological age of 2 years and 6 months old. The EEG showed mild cerebral dysfunction but no epileptiform discharge. The lower limb NCV study revealed bilateral sensorimotor polyneuropathies. Furthermore, diffuse cerebral and cerebellar atrophy were detected in his brain MRI. An increased signal intensity of the white matter was also discovered, indicating dysmyelination (or hypomyelination). On the other hand, the measurement of magnetic resonance spectroscopy (^1^H-MR spectroscopy, MRS) on the gray matter of the bilateral temporal lobes demonstrated that his N-acetylaspartate (NAA)/creatine (Cr) ratio was 1.09 on the right side and 1.18 on the left side. In addition, his choline (Cho)/Cr ratio was 0.85 on the right side and 0.87 on the left side. In this case, the NAA/Cr ratios of both sides were much lower than normal values (temporal gray matter: 2.026 ± 0.192) [17], while the Cho/Cr ratios were within the normal range (temporal gray matter: 0.929 ± 0.113) [17] (Figure 1B) as compared with previously published brain MRS studies [17,18,19].

Collectively, the above-described findings were similar to a previously reported neuroimaging study of CS. For example, Koob et al. reported that the NAA/Cr ratios of the white and gray matter were decreased in CS patients, and the Cho/Cr ratios of the white matter were decreased as well. In contrast, the Cho/Cr ratios of gray matter may be normal or decreased [20].

### 3.2. The Family Pedigree, Sanger Sequencing, the Conservation of the Amino Acid Sequences, and Domain Structure of CSB Protein

**Figure 2:**
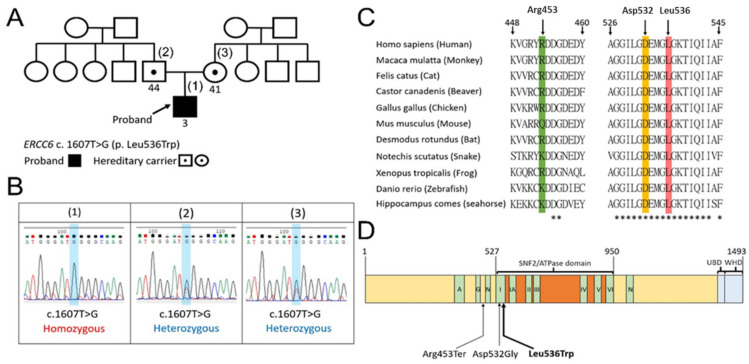
CS-associated missense mutations in *ERCC6* and their conservation. (**A**) Extended pedigree of this family. The black arrow points out the proband. The numbers below the squares and circles represent the age in year. (**B**) Sanger sequencing data of c.1607T>G (p.Leu536Trp) in the *ERCC6* gene of the proband and his parents. The heterozygous missense mutation c.1607T>G was identified in both of the parents. (**C**) Multiple sequence alignments of the indicated amino acids of CSB from mammals, birds, reptiles, and bony fish, containing conserved positions at Asp532 and Leu536. “*” indicates total identity of the amino acids aligned across these 11 species. (**D**) The Asp532Gly and Leu536Trp mutations are both located within the SNF2/ATPase domain motif I of CSB. A, acidic amino acid-rich region; G, glycine rich region; N, nuclear localization signal; UBD, ubiquitin-binding domain; WHD, winged-helix domain.

The proband is the only person who exhibited a development delay in his extended family and he is also the only child in the core family (Figure 2A). Sanger sequencing of c.1607T>G (p. Leu536Trp) in the *ERCC6* gene was performed on the proband and his parents. The heterozygous missense mutation c.1607T>G was identified in both of the proband’s parents, implying that his parents are hereditary carriers (Figure 2B).

Regarding the biologic function of the CSB protein, it contains a highly conserved ATP-binding domain [4]. We aligned multiple sequences of CSB containing the amino acids of interest to this work (i.e., Arg453, Asp532, and Leu536) from mammals to bony fishes, including *Homo sapiens, Macaca mulatta, Felis catus, Castor canadenis, Gallus gallus, Mus musculus, Desmodus rotundus, Notechis scutatus, Xenopus tropicalis, Danio rerio,* and *hippocampus comes* (Figure 2C). The Asp532Gly and Leu536Trp mutations are both located within the SNF2/ATPase domain motif I of CSB (Figure 2D). The total identity of the full-length CSB protein (aa: 1–1493) aligned across these 11 species is 35.7% (533/1493), while the total identity of the SNF2/ATPase domain (aa: 527–950) is 66.7% (283/424).

### 3.3. Predictive Three-Dimensional Models of CSB Protein

**Figure 3:**
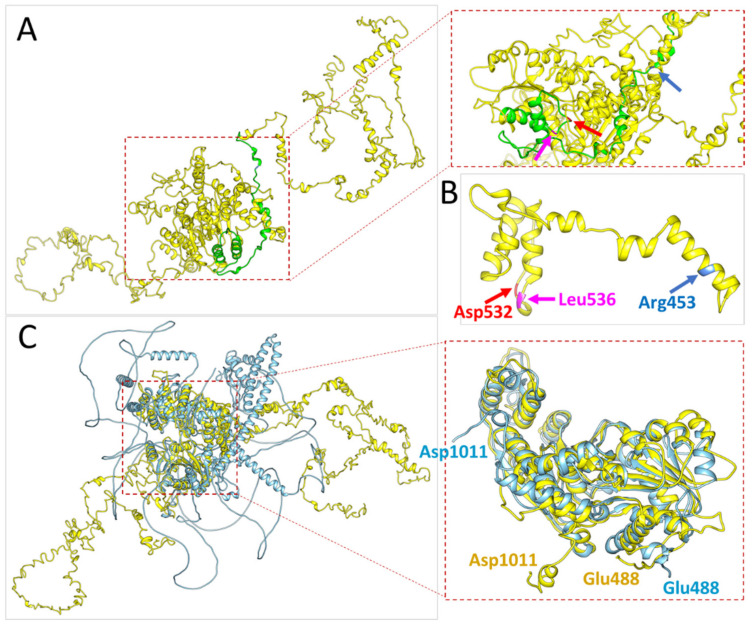
Three-dimensional structural models of CSB predicted by I-TASSER or Alphafold. (**A**) Partial length (aa: 440–550, green) and full-length (aa: 1–1493, yellow) with mutation sites labeling (I-TASSER). (**B**) Partial length (aa: 440–550, yellow) with the indicated mutations (I-TASSER). (**C**) Superposition of the two predicted CSB structures, superimposed length (aa: 488–1011) and full-length (aa: 1–1493), RMSD for Cα: 8.6 Å (yellow, I-TASSER; cyan, Alphafold).

We utilized the I-TASSER server [12] to predict the structures of the partial (aa: 440–550) and full-length (aa: 1–1493) sequences of CSB (Figure 3A). We labeled the mutation sites of amino acid found in our case as well as in the other two similar articles [7,8] at Arg453, Asp532, and Leu536 (Figure 3B). Meanwhile, we compared the predictive protein structures from I-TASSER with the other ones generated by the AlphaFold Protein Structure Database (https://alphafold.ebi.ac.uk/entry/Q03468, last updated on 1 July 2021) [13] by the RMSD method (Figure 3C). Through this method, we quantitatively measured the similarity of these two predictive structures. We found that the model from I-TASSER has an 8.6 Å Cα RMSD relative to the one from Alphafold (aa: 488–1011), which suggests a high degree of structural similarity between the two modeled protein structures. By using these two different models in our computational analyses of protein stability, we will increase the robustness of our findings.

**Figure 4:**
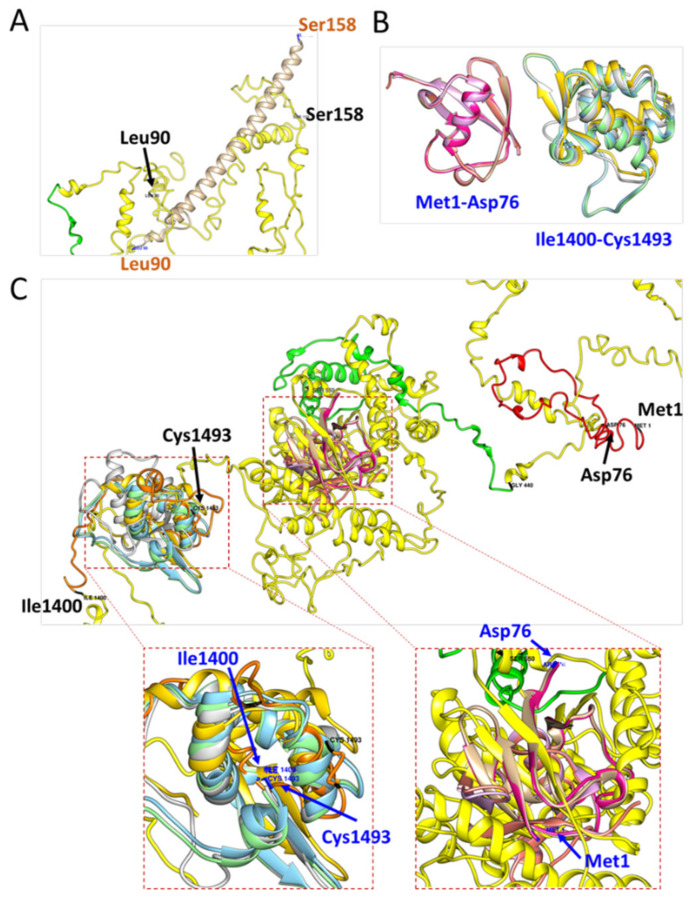
Three-dimensional models of CSB predicted by I-TASSER and compared with published structures. (**A**) Alignment of partial length (aa: 90–158) between the I-TASSER predicted model (yellow) and Newman’s model (beige, PDB: 4CVO). (**B**) Partial length (aa: 1–76; magenta) and (aa: 1400–1493; yellow-green), modified from Takahashi’s model (PDB: 6A6I). (**C**) Overview of the structure alignment between the I-TASSER predicted model (full-length) and Takahashi’s model. The region from amino acids 1400 to 1493 are highly overlapped with the I-TASSER model while the region from amino acids 1 to 76 does not.

Finally, we utilized the structure generated by I-TASSER to compare with the current known crystal structures of CSB from Newman’s model (aa: 90–158) (PDB code: 4CVO) and Takahashi’s model (aa: 1–76 and aa: 1400–1493), respectively [15] (PDB code: 6A6I). The model from I-TASSER neither superimposes to Newman’s model (aa: 90–158) (Figure 4A) nor to Takahashi’s model (aa: 1–76) (Figure 4B,C). However, it does superimpose to Takahashi’s model (aa: 1400–1493) to some extent (Figure 4B,C).

### 3.4. Computational Assessment of the Impact of CS-Associated Mutations on the Stability of CSB

The impact of CS-associated mutations on the stability of our predicted CSB structures was calculated using the BIOVIA Discovery Studio 2019 software. The Asp532Gly mutation destabilized both modeled structures. In contrast, the Leu536Trp mutation had no detectable effect on the I-TASSER model of CSB but destabilized the Alphafold’s model. The greatest destabilizing energy changes were shown for the modeled CSB structures containing both the Asp532Gly and the Leu536Trp mutations (Table 1). As for the calculation of the change in mutation, energy is normalized by wild-type CSB protein. On the other hand, the impact of the Arg453Ter mutation was not calculable by this method.

## 4. Discussion

Cockayne syndrome is divided into two categories by the mutations in the *ERCC6* and *ERCC8* genes. Approximately 62–75% of all CS cases had mutations in the *ERCC6* gene, which defined them as Cockayne syndrome B (CSB) [1,3]. There were 102 mutations found in *ERCC6* (50 homozygous and 52 heterozygous) and 37 mutations in *ERCC8* (23 homozygous and 14 heterozygous) [6]. Some of these mutations give rise to truncated CS proteins, but the severity of the phenotype presenting in the patients is not well correlated with their genotype. For instance, when analyzing homozygous mutations, we observed that the type of mutation or the affected region does not necessarily relate to the severity of CS [6].

### 4.1. The First Reported Homozygous Mutation Identified in the ERCC6 Gene (c.1607T>G, p.Leu536Trp)

In this study, the WES test of the proband identified the homozygous missense mutation c.1607T>G (p.Leu536Trp) in the *ERCC6* gene, which supported the clinical diagnosis of Cockayne syndrome B in our case. On the other hand, this missense mutation of *ERCC6* presenting in heterozygous form has been reported in two previous studies [7,8]. One study demonstrated Cockayne syndrome B in three Chinese sisters carrying compound heterozygous missense mutations of c.1595A>G (p.Asp532Gly) and c.1607T>G (p.Leu536Trp) in the *ERCC6* gene [7]. Another study identified Cockayne syndrome B in two Chinese brothers carrying compound heterozygous missense mutations of c.1357C>T (p.Arg453Ter) and c.1607T>G (p.Leu536Trp) in the *ERCC6* gene [8]. Intriguingly, our case is the first reported patient with a homozygous missense mutation c.1607T>G (p.Leu536Trp), which is inherited from both unaffected parents (Table 2).

### 4.2. Computational Modeling Results Strongly Suggest That a Homozygous Mutation in the ERCC6 Gene (c.1607T>G, p.Leu536Trp) Is Potentially a True Pathogenic Variant in Clinical Context

The ACMG defines this missense mutation in *ERCC6* as “likely pathogenic”, according to the interpretation from the Varsome database (https://varsome.com, accessed on 3 September 2021). In addition, this mutation has not been listed as a pathogenic variant in CSB by ClinVar Miner, which was updated on 31 July 2021 (https://clinvarminer.genetics.utah.edu, accessed on 3 September 2021). Contrarily, GenomAD v3.1.1 has interpreted this mutation as a pathogenic variant. To our knowledge, no homozygous missense mutation c.1607T>G (p.Leu536Trp) in the *ERCC6* gene has been reported to date. Even in the summary of the latest review article [6], this mutation is only reported in the heterozygous group. On the other hand, Leu536 is highly conserved across diverse species including human mammals, birds, reptiles, bony fishes, and seahorses. The Asp532Gly mutation also shows a similarly high level of conservation (Figure 2C,D). Consequently, we utilized computationally predicted CSB protein models to view the relative 3D spatial positions of Arg453, Asp532, and Leu536 (Figure 3A,B). Furthermore, we calculated the mutation energy change of these variants in our two predictive models, which demonstrated that the Asp532Gly mutation was potentially destabilizing in both models. However, the Leu536Trp mutation showed destabilizing effects in the Alphfold model (Table 1). Based on these findings, we suggest that this missense mutation c.1607T>G (p.Leu536Trp) in the *ERCC6* gene is a “pathogenic” mutation and this homozygous form indeed presents in the CSB case examined here. Moreover, c.1595A>G (p.Asp532Gly) is likely the same pathogenic in our interpretation with the evidence from a predictive crystal structure and mutation energy results despite the ACMG currently interpreting Asp532Gly as having uncertain significance.

### 4.3. A Founder Effect of the ERCC6 Gene Mutation (c.1607T>G, p.Leu536Trp) in the Southeast Coast of East ASIA

We investigated the allele frequency (AF) of the missense mutation c.1607T>G (p.Leu536Trp) in the *ERCC6* gene among different databases. In the Taiwan Biobank database (https://taiwanview.twbiobank.org.tw/browse38, accessed on 3 September 2021), the AF of this missense mutation is 0.001318 among 1517 Taiwanese individuals. In gnomAD v3.1.1 (https://gnomad.broadinstitute.org, accessed on 3 September 2021), the AF is 0.00002628 among 251,054 global papulations. Furthermore, the AF provided by the genomAD v3.1.1 with additional filtering in the east Asian population [21] is 0.0002627 (Table 2). The total six cases, including our proband, are consanguineously classified as Han people and live near the southeast coastal area of Mainland China that contains Shenzhen, Guangzhou, and Taiwan. We believe that this founder effect is related to the previous history of regional population migration in the southeast coastal area of east Asia [22]. Hence, this genetic variant has a higher AF around these areas and varies greatly from other populations and areas.

### 4.4. The Role of CSB in Neurodegeneration

The *ERCC6* gene encodes the CSB protein, and it consists of several domains, including an acidic amino acid-rich region, glycine-rich region, seven helicase ATP-binding motifs, and two nuclear localization sequences [5]. UV-light sensitivity is a hallmark of the CSB phenotype, which leads to DNA damage by the induction of cyclobutane pyrimidine dimers and 6-pyrimidine-4-pyrimidone products [5]. In normal cells, these lesions could be removed by the TCR, a sub-pathway of the NER. Accumulating evidence proved the dysfunction of this pathway in CSB-deficient cells, which indicated that CSB has an important role in the TCR [23,24]. Previous studies also revealed the role of CSB in the repair of oxidative DNA damage, mainly by BER [25,26]. This function is consistent with cellular studies that demonstrated the accumulation of oxidative DNA damage in CSB-deficient fibroblasts isolated from CS patients after oxidative stress exposure [27].

### 4.5. Mutations in the ATPase Motif of CSB Inactivate Its ATPase Activity and Ultimately Its Function

The ATPase motifs in CSB provides the energy needed for its association with chromatin. Notably, mutations in motif I and II, named “Walker A” and “Walker B,” respectively, can completely inactivate the ATPase activity of CSB [4]. The presence of a point mutation in the Walker A motif of CSB may impair its ability to interact with ATP, which would clearly prevent its ability to hydrolyze ATP and therefore function in cells, as was previously demonstrated in human fibroblasts [28] In another study, a single amino acid substitution in the highly conserved residues of ATPase motifs Ia, III, V, and VI abolished the function of the CSB protein in the survival of patient-derived fibroblasts, RNA synthesis recovery, and apoptosis after UV exposure [29]. Furthermore, our study showed that the total identity of the SNF2/ATPase domain (aa: 527–950) across 11 species is 66.7% (283/424 amino acids) (Figure 2D). These above studies collectively suggest that the pathogenicity of the missense mutation, Leu536Trp, as well as the Asp532Gly mutation, which are both found in ATPase motif I, may cause the loss of the ability of CSB to repair damaged DNA, leading to neurodegeneration and mitochondrial dysfunction in CS patients [30]. This idea is further supported by a *Caenorhabditis* elegans model that demonstrates the role of endogenous DNA damage as a driving factor of CS-related neuropathology and underlines the neuronal and mitochondrial aberrations observed in the disease [31].

### 4.6. Potential Treatments for CS Patients

To date, no specific treatment to cure or slow the progression of CS exists. Physical therapy, personal education, prevention of sunlight exposure, cochlear implants for hearing loss [32], feeding gastrostomy tube for malnutrition, medication for tremor [33], etc. may be helpful if needed [1]. Intriguingly, some studies revealed that autophagic stimulators may be promising therapies for CS [30,34]. For example, previous studies found the decreased colocalization of proteins involved in mitophagy (i.e., LC3, P62, and ubiquitin), the process by which mitochondria are selectively degraded in response to stress. This finding explained the increased mitochondrial content, increased membrane potential, and increased free radical production observed in CSB-deficient cells [34]. Rapamycin is thought to be neuroprotective, and it is able to decrease glial activation; therefore, it could secondarily attenuate some of the neurological features of CS [35,36]. Consistent with this hypothesis, CSB was shown to induce mitophagy in response to oxidative stress by sensing the presence of damaged mitochondrial DNA [34]. Pharmacological modulators of autophagy such as rapamycin or lithium chloride could decrease the bioenergetic phenotype of CSB-deficient cells, which may represent potential treatment options to counteract accelerated aging phenotypes and increase longevity in CS patients [34,37].

## 5. Conclusions

In summary, we described a Taiwanese boy with Cockayne syndrome presenting novel homozygous missense mutation c.1607T>G (p.Leu536Trp) in the *ERCC6* gene, which has not been reported before. Furthermore, we suggest that the CS-associated Asp532Gly and Leu536Trp mutations are both pathogenic variants, while the Arg453Ter mutation has been defined as a pathogenic variant in the database. These findings provide important objective evidence to suggest that the potential pathogenicity of these missense mutations in Cockayne syndrome B.

## Figures and Tables

**Table 1:** The effect of CS-associated mutations on the stability of human CSB (488–1011) *.

Model	I-TASSER		AlphaFold	
Mutation	Mutation Energy (kcal/mol)	Predicted Effect	Mutation Energy (kcal/mol)	Predicted Effect
Asp532→Gly532	3.56	Destabilizing	5.8	Destabilizing
Leu536→Trp536	0.18	Neutral	0.98	Destabilizing
Asp532→Gly532+ Leu536→Trp536	4.01	Destabilizing	6.99	Destabilizing

* Mutation on the stability of human CSB protein was calculated using the two predicated models from I-TASSER and AlphaFold.

**Table 2:** Comparison with two other studies associated with identical *ERCC6* c.1607T>G (p.Leu536Trp) missense mutation in Cockayne Syndrome B.

	Zhonghua Er Ke Za Zhi (Zhizi Zhou, 2016) [8]		PLoS One (Yu et al., 2014) [7]		Our Patient
Case Number	2		3		1
Mutation	Compound heterozygous		Compound heterozygous		Homozygous
GRCh37	Chr10:50732119	Chr10:50708662	Chr10:50708674	Chr10:50708662	Chr10:50708662
dbSNP	rs121917902	rs774175886	rs752712823	rs774175886	rs774175886
DNA change	c.1357C>T	c.1607T > G	c.1595A>G	c.1607T>G	c.1607T>G
Amino acid change	p.Arg453Ter	p.Leu536Trp	p.Asp532Gly	p.Leu536Trp	p.Leu536Trp
Allele frequency (Taiwan) ^1^	NA	0.001318	NA	0.001318	0.001318
Allele frequency (Global) ^2^	0/478 (ALFA^3^ Project)	0.00002628 (GenomAD v3.1.1)	0.000003983 (GenomAD v2.1.1)	0.00002628 (GenomAD v3.1.1)	0.00002628 (GenomAD v3.1.1)
Allele frequency (East Asian) ^3^	NA	0.0002627 (GenomAD v3.1.1)	NA	0.0002627 (GenomAD v3.1.1)	0.0002627 (GenomAD v3.1.1)
ACMG Classification	Pathogenic	Likely Pathogenic	Uncertain Significance	Likely Pathogenic	Likely Pathogenic
PMID	26791926		25463447		Non-Applicable

^1^ Taiwan Biobank: https://taiwanview.twbiobank.org.tw/index. ^2^ GenomAD: https://gnomad.broadinstitute.org/. ^3^ ALFA, Allele Frequency Aggregator (https://www.ncbi.nlm.nih.gov/snp/docs/gsr/alfa (accessed on 3 September 2021).

## Data Availability

All the data are contained within the article.
